# Supplementary material for: Reliability-enhanced data cleaning in biomedical machine learning using inductive conformal prediction
Source: PLoS Comput Biol. 2025 Feb 13;21(2):e1012803. doi: 10.1371/journal.pcbi.1012803 (PMC11870354; doi:10.1371/journal.pcbi.1012803)
Supplement: S1 Text [file pcbi.1012803.s001.docx]

**S1 Text**

**Cleaning Process Variation with Different Hyperparameters**

To investigate the influence of the CPSC hyperparameters on the data cleaning process, we plotted the changes in wrongly labeled data detected with varying Δ and T in the S4 and S5 Figs. We have observed that increasing Δ reduces the number of wrongly labeled data detected, particularly when more training labels are permuted (more than 40% label permutation in the proper training set). For the temperature parameter, more detected wrongly labeled data occurred when the parameter is set to 10.

We hypothesize that with increasing Δ, the noise attenuation function of the CPSC has been boosted, together with more information in the data lost after the soft thresholding. As a result, CPSC tends to be less certain in determining wrongly labeled data. For the temperature parameter, a higher temperature (100) can lead to a more flattened distribution of predicted probability after soft-max layer, which can lead the models to be less confident in telling wrongly labeled data. When T is set too low, the CPSC tends to output more spiky probabilities when the class-related information is clearly represented in the features. When the dataset becomes noisy, the CPSC may be overfit to the noise and the spiky distribution output by CPSC may not be informative enough to calibrate the p-values for the detection of wrongly labeled data. This behavior has been observed in our previous study when CPSC was proposed.

**COVID-19 Data Collection and Preprocessing**

COVID-19 data (n=2,113) in this study were collected from 40 hospitals in China from December 27, 2019, to March 31, 2020. Patients’ selection followed the inclusion criteria: (a) RT-PCR confirmed positive severe acute respiratory syndrome coronavirus (SARS-CoV-2) nucleic acid test; (b) baseline chest CT examinations and laboratory tests on admission; (c) short-term prognosis information (discharge or admission to ICU).

Clinical, lab, and radiomics data for each patient included:

1) Clinical data based on electronic Health Records (EHR): (a) demographics: age and gender; (b) comorbidities: coronary heart disease, diabetes, hypertension, chronic obstructive lung disease (COPD), chronic liver disease, chronic kidney disease, and carcinoma; (c) clinical symptoms: fever, cough, myalgia, fatigue, headache, nausea or vomiting, diarrhea, abdominal pain, and dyspnea on admission. To extract the clinical data from the free-text EHR in Chinese, we developed a rule-based language processing algorithm. Firstly, the clinical descriptions are segmented from EHR by splitting the paragraphs with subtitles. Then, we established a keyword list containing all the descriptions associated with a particular clinical feature, such as 'fever', 'cough'. Simply using a regular expression to match keywords is not practical. An example of the clinical description is: "This patient had fever and cough three days ago, and he had no diarrhea or vomiting, and today he is transmitted to this hospital without fever". Since the symptoms can progress/recover, the same keywords have different meanings with negation and thought groups. Therefore, we designed a voting rule to extract the clinical data: by breaking down the clinical descriptions with commas, the keywords can be matched into several thought groups. In each thought group, the frequency of target keywords is recorded, with a default value of zero. If a thought group begins with negation (such as 'no', or 'did not find'), the keywords appeared to vote zero to their encoding values; Otherwise, the keywords appeared to vote one. The votes are summed up and compared with zero, and then the sum will convert into a Boolean value and be viewed as the encoded value for a specific clinical feature. With the voting rule, the negative appearance of a keyword can be distinguished, and the sum of votes can provide more information for further study as it indicates the frequency of a symptom.

2) Laboratory test: blood routine, coagulation function, blood biochemistry, infection-related biomarkers. (a) blood routine: white blood cell (WBC) count (*10^9^/L), neutrophil count (*10^9^/L), lymphocyte count (*10^9^/L), platelet count (*10^9^/L), and hemoglobin (g/L); (b) coagulation function: prothrombin time (PT) (s), activated partial thromboplastin time (aPTT) (s), and D-dimer (mg/L); (c) blood biochemistry: albumin (g/L), alanine aminotransferase (ALT) (U/L), aspartate Aminotransferase (AST) (U/L), total bilirubin (mmol/L), serum potassium (mmol/L), sodium (mmol/L), creatinine (μmol/L), creatine kinase (CK) (U/L), lactate dehydrogenase (LDH) (U/L), α-Hydroxybutyrate dehydrogenase (HBDH) (U/L);

(d) infection-related biomarkers: C-reactive protein (CRP) (mg/L). Patients took laboratory tests on the date of admission in the training set, validation set, and Variants test set, while patients in the Huoshenshan Hospital subset received laboratory tests within two days after admission due to the centralized outbreak in Wuhan and the limited medical resources. To alleviate missing values that occurred in records, we applied median imputation on the lab data when a missing rate was <50%, which has been validated effective in the previous study [1, 2]. Each inpatient received laboratory tests within 48h after admission and only clinical data on or prior to the date of the CT were used for prediction.

3) CT radiomics: Patients took baseline CT scans within three days after admission. It should be mentioned that Chinese guidelines for the diagnosis and treatment of novel coronavirus infection recommend the CT imaging features as one of the criteria for clinical classification, the treatment chosen, and the discharge criteria for hospitalized patients. Chest CT scans were performed using > 16 slice multidetector CT scanners (Aquilion ONE / Aquilion PRIME / BrightSpeed / BrightSpeed S / Brilliance 16 / Brilliance 64 / Discovery CT750 HD / eCT / Fluorospot Compact FD / HiSpeed Dual / iCT 256 / Ingenuity CT / Ingenuity Flex / LightSpeed VCT / LightSpeed 16 / NeuViz 16 Classic / Optima CT520 Series / Optima CT540 / Optima CT680 Series / ScintCare CT 16E / Sensation 64 / SOMATOM Definition AS+ / SOMATOM Definition Flash / uCT 510) without use of iodinated contrast agents. To minimize motion artifacts, patients were asked to hold their breath, then axial CT images were acquired during end-inspiration. The CT scan protocols were as follows: tube voltage, 100-120 kV; effective tube current, 110-250 mAs; detector collimation, 16-320 * 0.625-2.5 mm; slice thickness, 0.625-2.5 mm; pitch, 0.8-1.375. The CT images were reconstructed by iterative reconstruction technique if possible based on the raw data. A commercial deep-learning AI system (Beijing Deepwise & League of PhD Technology Co. Ltd) was first used to detect and segment the pneumonia lesion, and two radiologists (Q.M.X. and C.S.Z.) checked the results of the automatic segmentation. Then, pyradiomics (v3.0) running in the Linux platform was adopted to extract radiomic features (1652 features per lesion). Next, for a given patient and for each radiomic feature, we summarized the distribution of the feature values across all the lesions for the patient by several summary statistics (mean, median, standard deviation, skewness, the first quartile, the third quartile) and the number of lesions. Finally, a total of 9913 quantitative radiomic features were extracted from CT images for each patient.

**Reference**

1. Xu Q, Zhan X, Zhou Z, Li Y, Xie P, Zhang S, et al. AI-based analysis of CT images for rapid triage of COVID-19 patients. NPJ digital medicine. 2021;4(1):1–11.
2. Xu C, Xu Q, Liu L, Zhou M, Xing Z, Zhou Z, et al. A tri-light warning system for hospitalized COVID-19 patients: Credibility-based risk stratification for future pandemic preparedness. European Journal of Radiology Open. 2024;13:100603.
